# The multifaceted virulence of adherent-invasive *Escherichia coli*

**DOI:** 10.1080/19490976.2023.2172669

**Published:** 2023-02-05

**Authors:** Sarah Mansour, Tahreem Asrar, Wael Elhenawy

**Affiliations:** a Department of Medical Microbiology & Immunology, Faculty of Medicine & Dentistry, University of Alberta, Canada; b Division of Gastroenterology, Department of Medicine, Faculty of Medicine & Dentistry, University of Alberta, Edmonton, Alberta, Canada; c Li Ka Shing Institute of Virology, Canada; d Women and Children’s Health Research Institute, Edmonton, Alberta, Canada; e Antimicrobial Resistance, One Health Consortium - Edmonton, AB, Canada

**Keywords:** Inflammation, Crohn’s disease, adherent-invasive*E. coli*, metabolic adaptation, immune evasion

## Abstract

The surge in inflammatory bowel diseases, like Crohn’s disease (CD), is alarming. While the role of the gut microbiome in CD development is unresolved, the frequent isolation of adherent-invasive *Escherichia coli* (AIEC) strains from patient biopsies, together with their propensity to trigger gut inflammation, underpin the potential role of these bacteria as disease modifiers. In this review, we explore the spectrum of AIEC pathogenesis, including their metabolic versatility in the gut. We describe how AIEC strains hijack the host defense mechanisms to evade immune attrition and promote inflammation. Furthermore, we highlight the key traits that differentiate AIEC from commensal *E. coli*. Deciphering the main components of AIEC virulence is cardinal to the discovery of the next generation of antimicrobials that can selectively eradicate CD-associated bacteria.

## Introduction

The role of the gut microbiome in promoting human health is indubitable. It extends from providing colonization resistance against invading microbes to expanding the digestive ability of the host and regulating its immune functions. In this regard, the metabolites produced by the gut microbiome are known to regulate immune homeostasis and promote mucosal integrity.^1^ This intertwined connection between gut health and the microbiome motivated the investigation of their role in driving inflammatory bowel diseases like Crohn’s Disease (CD). In addition to negatively impacting the quality of life, CD imposes a significant burden on the healthcare system due to its recurrent fulminant episodes that can lead to repeated hospitalization throughout the lifetime of the patient.^2^ The increasing incidence of CD in the Western hemisphere since 1960 suggests that the factors predisposing for the disease are surging.^3^ Genome-wide association studies have identified over 160 susceptible loci that may contribute to the increased risk of developing inflammatory bowel diseases (IBD) like CD.^4–7^ This includes many loci that are involved in sensing and responding to microbes, suggesting that the microbiome is implicated in CD etiology. In accord with this, the contribution of host genetics to CD development was found to be more prominent in the pediatric population compared to adults, which sheds light on the role of the environmental factors as disease modifiers. Additionally, the concordance of CD among monozygotic twins was found to be approximately 50%,^8^ confirming that genetic and non-genetic risk factors converge to propel disease development. While CD is marked by a dysregulated immune response to the gut microbiome, the role of specific microbes in promoting these pathological interactions remains unclear.

Due to its contribution to the development of intestinal granulomatous diseases in ruminants, *Mycobacterium avium* subspecies *paratuberculosis* (MAP) was proposed to be the causative agent of CD.^9–11^ Although detected by PCR in the intestinal tissues of CD patients, MAP was also found in the biopsies collected from the control group.^12^ While these bacteria are commonly associated with livestock, no increase in CD incidence among farmers was reported, a trend that would be expected for a zoonotic pathogen.^13^ Additionally, anti-Mycobacterial therapies did not improve remission rates among CD patients.^14^ Other studies employed immunostaining of clinical samples to investigate the potential contribution of bacterial pathogens to CD.^15^ Using biopsies collected from 21 patients, Liu *et al* showed a correlation between CD and *Escherichia coli, Listeria monocytogenes*, and *Streptococcus spp*. However, another study ruled out a role for *Listeria* in disease development, since immunostaining revealed no preferential abundance of these bacteria in the CD gut.^16^ Of note, it is unclear whether the inconsistency between studies in defining the CD-associated microbial community reflects the presence of different subtypes of the disease.

In the quest to isolate the gut microbes that are associated with CD, the late Dr. Darfeuille-Michaud identified a new pathogroup of bacteria called adherent-invasive *Escherichia coli* (AIEC).^17^ The likelihood of isolating these bacteria from CD patients is approximately six-times higher than from healthy individuals, suggesting a role in disease development.^18,19^

Members of the AIEC group can invade the intestinal epithelial cells and replicate inside macrophages similar to entero-invasive *E. coli* strains. Nevertheless, AIEC strains lack several prominent virulence factors that are known to promote invasion, such as type III secretion system.^20,21^ Comparative genomics revealed a significant strain divergence among the AIEC group and a lack of a defining genetic blueprint, making this pathovar genetically inextricable from commensal *E.coli*.^20,22,23^ While phylogenetic analysis suggests that AIEC strains belong primarily to the B2 clade,^20,24–26^ a different phylogroup distribution was reported for the strains isolated from the pediatric patient population.^27^ Interestingly, this remarkable genetic heterogeneity was also observed within the AIEC populations isolated from individual patients.^24,27^ Similarly, diversification marks the evolutionary response of AIEC to host selection in CD infection models.^28^

To this end, the identification of AIEC relies on time consuming and non-standardized phenotypic assays *in vitro*.^23^ Although the evolutionary origins of AIEC are nebulous, several groups demonstrated that these bacteria could trigger inflammation in the preclinical models of colitis; thus, fulfilling Koch’s postulates in the susceptible hosts.^29–32^ However, the intricacies of AIEC-host interactions, and how these pathobionts evolved differently from commensals, remain unknown. Unlike commensals, the pathogenic potential of pathobionts manifests under certain host conditions.^33^ In this article, we review the factors that promote the virulence of AIEC in the host environment. We discuss how AIEC strains can modulate the immune responses to persist in the gut. Additionally, we highlight several examples of the metabolic versatility of this group of bacteria, which is key to their *in vivo* fitness. While there are other bacteria that have been implicated in CD, their contribution to disease development is beyond the scope of this review.

## The main features of AIEC pathogenesis

The ability of AIEC strains to adhere to and invade epithelial cells distinguishes them from commensal *E. coli*. This is mediated by an arsenal of virulence factors including the adhesin of type I pili, FimH, which can bind to Carcinoembryonic Antigen-related Cell Adhesion Molecules 6 (CEACAM6) receptor.^34,35^ Interestingly, these receptors are upregulated in the ileal lesions of CD patients, possibly influencing the trajectory of AIEC adaptive evolution.^34^ This is evident by the enhanced CEACAM6-binding ability of the FimH alleles within the AIEC group relative to their homologues in commensal *E. coli*.^36^ While these allelic variations are not always captured by comparative genomics studies, they highlight the different evolutionary trajectories of AIEC and commensals. In line with its role in promoting host colonization, blocking FimH using drugs^37^ or glycoconjugates^38^ curtailed bacterial adhesion to the intestinal epithelium and ameliorated inflammation. In addition to FimH, OmpC was shown to be important for the ability of AIEC to adhere to the intestinal cells. Although its binding target in the host cells remains unknown, OmpC was upregulated *in vitro* by high osmolarity similar to that encountered by bacteria in the gut, suggesting a role for this protein in promoting *in vivo* fitness.^39^ In line with this, antibodies targeting OmpC were significantly more abundant in the sera of CD patients relative to healthy individuals, and could be used in combination with other antibodies as reliable markers for IBD.^40^ ChiA is another adhesin that mediates the ability of AIEC to attach firmly to the intestinal epithelial cells.^41^ Similar to other chitin-binding domains in *Serratia marcescens* and *Vibrio cholera*,^42,43^ ChiA binds to Inducible Chitinase 3-Like-1 (CHI3L1), an enzymatically inactive *N-*glycosylated chitinase that is overexpressed in the colon during inflammation. Interestingly, AIEC strains harbor ChiA variants with 5 specific polymorphisms that confer increased binding to CHI3L1 compared to those in commensal K-12 strains,^41^ supporting the role of this adhesin in promoting the virulence of CD-associated pathobionts.

Along with their main function of mediating swimming motility, flagella also play a key role in facilitating cell adhesion and invasion by AIEC.^28,44^ Compared to commensal *E. coli*, AIEC strains are hyperflagellated, which is congruent with their superior ability to penetrate the intestinal crypts.^28^ Interestingly, deleting the flagellin-encoding gene in AIEC results in low piliation due to the inversion of a genetic switch in the *fim* promoter, suggesting a regulatory crosstalk between type I pili and flagella.^45^ Given that both surface organelles promote cell adhesion, it is possible that the elements controlling their expression were wired during reductive evolution to generate a regulatory node that can respond to common host cues. Together, these data highlight the ability of AIEC strains to deploy multiple adhesins that can facilitate host cell contact. Nonetheless, such contact is contingent on the ability of these bacteria to overcome the mucus layer covering the epithelial cells. While flagella propel AIEC across the mucin, the ability of these bacteria to secrete mucinolytic enzymes, like Vat, further promotes mucosal invasion. This protease belongs to the SPATE family (Serine Protease Autotransporters of the Enterobacteriaceae) and is significantly overrepresented in CD-associated *E. coli* relative to commensals.^46^ Moreover, Vat is upregulated *in vitro* under conditions that mimici the ileum, highlighting the influence of the anatomical microenvironments on virulence gene expression.^46^

In addition to cell adhesion, AIEC strains are known for invading the host cells despite lacking type III secretion systems, which are commonly used by enteric pathogens to trigger their uptake.^20,21^ This can be explained by the invasins harbored within AIEC strains. This includes IbeA, an outer membrane invasin that interacts with host receptors such as vimentin. IbeA was found to be crucial for the ability of AIEC to invade the host cells and colonize the murine gut.^47^ Interestingly, these findings were confirmed by a recent analysis of the *in vivo* transcriptome of AIEC, which revealed that *ibeA* is upregulated during the early phase of host infection.^48^ Taken together, these data suggest that the fitness of AIEC in the CD environment is highly reliant on their access to the epithelial cells.

The predilection of AIEC to invade the gut epithelia could be explained by several factors. First, invading the epithelial cells and establishing an intracellular niche is an efficient mechanism to evade the phagocytic immune cells in the gut. This is further facilitated by the defects in the microbial sensing mechanisms, which are common among CD patients.^49^ Second, the epithelial microenvironment provides AIEC with access to mucosal metabolites that are beyond the reach of the autochthonous microbes residing in the gut lumen. For example, AIEC can utilize some of the host-derived amino acids only when they are in close association with the epithelial cells.^50^ Thus, the egress of the AIEC population, or a subpopulation, from the gut lumen toward a microenvironment with less residing microbes allows them to thrive in a less competitive niche. Indeed, the intracellular lifestyle of AIEC is not limited to epithelial cells. Since their discovery, AIEC strains were recognized for their ability to replicate within macrophages at comparable levels to that of known intracellular pathogens like *Shigella* and *Salmonella*.^51^ However, these pathogens hijack the host defense responses using their type III secretion systems, which are absent in AIEC. This suggests that AIEC strains employ unique mechanisms to establish a replicative niche inside the host cells.^21,22^ Data from different groups suggest that the stress response is key to the survival of AIEC in macrophages. For example, the stress protein, HtrA, is required by AIEC to survive in the hostile phagolysosomal environment, potentially through its housekeeping role in degrading misfolded proteins. Interestingly, *htrA* was upregulated by AIEC, but not commensal *E. coli*, inside macrophages, further confirming the improved ability of CD-associated pathobionts to persist in hostile host environments.^52^ Furthermore, recent work revealed that some AIEC strains are able to assemble intracellular biofilms, which fosters their ability to replicate inside macrophages.^53^ The authors used transcriptomics to track gene expression by the intracellular bacteria. Expectedly, the genes involved in the response to oxidative stress and acidic pH are upregulated by the intracellular bacterial population. This includes the SOS response that is triggered by the genotoxic damage encountered by AIEC inside the vacuole. Additionally, the survival of AIEC inside the phagolysosomal compartment required an intact stringent response, with the latter mediating the emergence of a quiescent subpopulation that is tolerant to antibiotics.^54^ Similarly, persister cell formation by the enteric pathogen, *Salmonella enterica*,^55^ was previously reported suggesting that this is a common trait among intracellular pathogens. However, members of the AIEC pathovar display significant variability in cell invasion and intracellular replication.^22^ Thus, a universal role for stringent and SOS responses among members of the AIEC group remains to be demonstrated.

## Immune evasion by AIEC

The gut environment is equipped with several defensive mechanisms that impede the luminal bacteria from breaching the mucosal barrier. In addition to the mucus layer that physically separates most members of the gut microbiome from the underlying epithelial cells, phagocytes such as macrophages and conventional dendritic cells are continuously surveilling the mucosa for invading pathogens.^56^ Therefore, it is not surprising that some of the risk alleles associated with CD encompass key components of the immune system that are required for sensing and eliminating bacteria.^49^ This includes autophagy, an important housekeeping process that is required for recycling different cellular organelles and eliminating intracellular pathogens.^57^ Missense variants of key autophagy genes, ATG16L1 and IRGM, are associated with an increased risk of CD.^6,58^ Additionally, the EIF2AK4-EIF2-ATF4 pathway, which regulates autophagy, is suppressed during CD-associated inflammation, causing impaired eradication of intracellular bacteria^59^ . In line with the role of autophagy in innate immunity, many pathogens have acquired the ability to hijack this pathway during their evolution.^60^ In this regard, AIEC strains suppress autophagy by increasing the expression of the host regulatory microRNA molecules, *Mir30c* and *Mir130a*. Upon overexpression, *Mir30c* and *Mir130a* downregulate ATG16L1 and ATG5, which are essential components of the autophagy response. During the early stages of autophagy, ATG16L1 will form a complex with ATG5 and ATG12, mediating membrane expansion to form the autophagosome.^61^ Clinical data support the role of *Mir30c* and *Mir130a* in modulating autophagy during inflammation as both microRNA molecules were overexpressed in the ileal biopsies obtained from CD patients. While the authors did not demonstrate that these tissues were infected with AIEC, the data highlight the potential of CD-associated bacteria to curtail autophagy, promoting their persistence in the intracellular environment.^62^

In addition to autophagy, the secretion of antimicrobial peptides by Paneth cells in the gut is one of the pillars of innate mucosal immunity.^63^ By disrupting the integrity of bacterial membranes, antimicrobial peptides limit the numbers of microbes growing at the intestinal epithelial surface. Some of the risk alleles associated with CD were found to affect the functions of Paneth cells. For example, murine models lacking NOD2, a common target for polymorphism in CD patients, display lower levels of α -defensin secretion compared to wild-type mice.^64^ This reduction is dependent on the microbiome as *Nod2 ^−/−^* mice co-housed with wild-type littermates displayed similar levels of α -defensin expression, highlighting the role of the microbiome in disease development.^65^ Furthermore, the clinical data suggest that the secretion of antimicrobial peptides is altered during CD. When the expression of antimicrobial peptides was compared between CD patients and healthy controls, lower levels of α -defensins were detected in the biopsies obtained from the patients presenting ileal disease. Interestingly, the low expression of α -defensins correlated with NOD2/CARD15 mutations, supporting the role of NOD2 in antimicrobial peptide production.^66^ Similar to ⍺-defensins, β-defensins like hBD-1,2,3 are differentially expressed in the guts of CD patients relative to the healthy population. While lower levels of hBD1 were detected in CD biopsies relative to healthy tissues, the opposite trend was observed for hBD 2 and 3.^67^ These data suggest that Paneth cell functions are modulated rather than diminished during CD. It is possible that the changes in antimicrobial peptide composition during CD contribute to the dysbiosis that characterizes the disease. In this regard, the predominant host defense peptides in the CD environment will select for the resistant bacterial strains, while driving the demise of those that are susceptible. In line with this hypothesis, AIEC strains are highly resistant to α- and β-defensins.^68^ This is dependent on the presence of outer membrane protease, OmpT, and other proteins that belong to Mig-14 family, known to mediate resistance to antimicrobial peptides^68,69^ . Interestingly, the resistance of AIEC to host defense peptides is required to exacerbate the inflammation caused by acute infectious gastroenteritis, a known risk factor for CD.^70^ Using a polymicrobial infection model, Small *et al* showed that AIEC can worsen the inflammation triggered by enteric pathogens, like *Salmonella* typhimurium and *Citrobacter rodentium*. Unlike the wild-type AIEC, an isogenic mutant susceptible to host defense peptides showed less morbidity when the animals were co-infected with *S*. typhimurium,^71^ confirming that antimicrobial peptide resistance is key for the ability of CD-associated pathobionts to aggravate inflammation.

## Metabolic adaptation of AIEC

**Figure 1.**
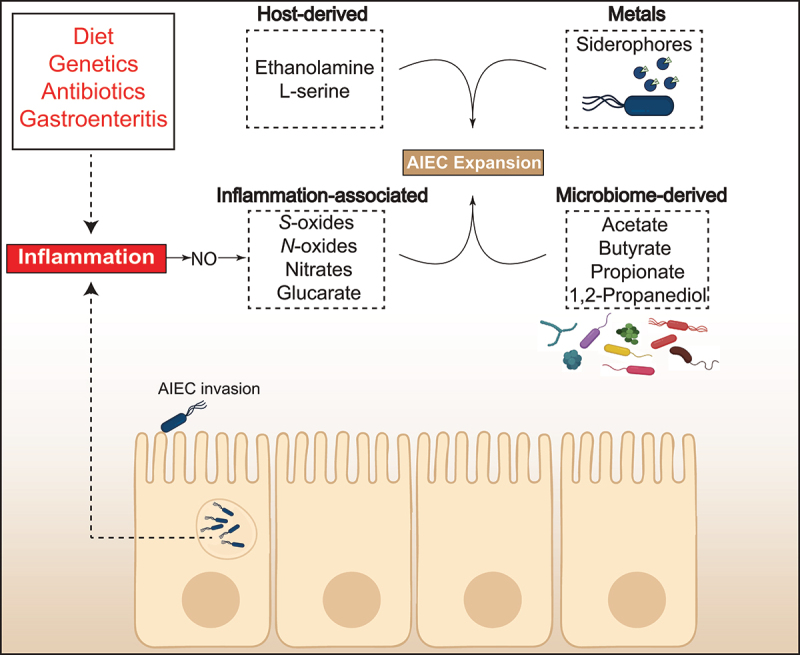
Examples of the key metabolites utilized by AIEC in the gut environment.

The ability of pathogens to utilize the metabolites available in the gut during inflammation is key to their expansion at the expense of the healthy microbiome.^72,73^ Similar to several enteric pathogens, the consumption of inflammation-associated metabolites by AIEC contributes significantly to their success in colonizing the gut (Figure 1). During inflammation, there is an increased production of nitric oxide, which can react with superoxide to generate peroxynitrite. Due to its instability, peroxynitrite can decompose into nitrate, or reacts with organic sulfides and tertiary amines, releasing *S*-Oxides and *N*-Oxides into the gut.^74^ Unlike strict anaerobes, facultative anaerobes, including members of family Enterobacteriaceae, can use nitrates, *S*-Oxides and *N*-Oxides as terminal electron acceptors during respiration.^75^ This provides Enterobacteriaceae, like *E. coli*, a metabolic advantage in the inflamed gut. Indeed, nitrate utilization fuels the expansion of AIEC during inflammation.^76^ An AIEC mutant deficient in utilizing nitrate as a terminal electron acceptor displayed lower fitness in the host environment compared to wild-type isogenic strain. Similarly, the consumption of oxidized sugars, like glucarate and galactarate, is instrumental for AIEC to thrive in the gut during the inflammation that ensues from antibiotic administration, a common treatment to manage CD flares.^76^ These results were confirmed using a high-resolution *in vivo* genetic screen that described the fitness landscape of AIEC in the gut.^48^ Using a combined genomics approach, genes that are essential for host colonization and are expressed by AIEC early during infection were mapped. Indeed, this analysis confirmed that metabolic adaptation is central to the ability of AIEC to thrive in the gut.^48^

To investigate the metabolic response of AIEC to inflammation, Kitamoto *et al* mapped the bacterial transcriptome during severe colitis.^77^ Many pathways involved in amino acid metabolism were specifically upregulated during inflammation compared to normal intestinal conditions. This includes the pathways for utilizing L-serine, which was found to be critical for the ability of AIEC to expand in the inflamed intestine and to exacerbate colitis.^77^ This fitness advantage is contingent on the availability of L-serine in the gut. Accordingly, the deprivation of dietary L-serine tied the fitness of AIEC to the presence of mucolytic symbionts that can degrade the host mucins to liberate this amino acid.^50^ Importantly, these studies highlight how diet can modulate the behavior of AIEC *in vivo*. Similarly, a Western diet that is high in fat and sugar promotes the expansion of AIEC in the gut.^78,79^ Interestingly, the low fiber content of Western diet was shown to be the main driver of AIEC expansion compared to high fat and sugar content, uncovering the potential of dietary intervention to combat the CD-associated pathogens.^79^

Other gut metabolites consumed by AIEC strains include fucose and its fermentation product, 1,2-propanediol.^80^ Using the propanediol dehydratase, PduC, *E. coli* can convert 1,2-propanediol to propionate, which can be further metabolized to pyruvate to be oxidized in the tricarboxylic acid cycle. Comparative genomics revealed that the *pdu* operon is more enriched in the genomes of AIEC relative to commensal *E. coli*.^81^ In agreement with its role in promoting AIEC expansion in the gut, PduC is upregulated *in vitro* in the presence of bile salts, commonly present in the intestine.^80^ Similarly, bile salts induced the *eut* operon, required for AIEC to utilize ethanolamine as a nitrogen source.^80^ Ethanolamine is released from phosphatidylethanolamine, which is highly abundant in the cell membranes of the host and its microbiome. Using clinical isolates, Zhang *et al* showed that AIEC strains can utilize ethanolamine as both carbon and nitrogen sources. Of note, this was enhanced by the presence of other inflammation-associated metabolites.^82^ Additionally, the authors investigated how inflammation-associated metabolites can influence global gene expression in AIEC, showing that some metabolites can upregulate virulence genes. However, it is unclear if this *in vitro* analysis approximates the transcriptomes of bacteria *in vivo*, given the complexity of the metabolic landscape in the gut. Nevertheless, other studies suggest that ethanolamine utilization supports AIEC blooms in the gut. For example, lipid precursors of ethanolamine, *N*-acylethanolamines, are elevated in the gut of IBD patients and preclinical models of colitis, highlighting the importance of this metabolite in fueling the expansion of CD-associated bacteria.^83^ These results were further confirmed by a recent study that mapped the *in vivo* transcriptome of AIEC, showing that several ethanolamine utilization genes are upregulated by these bacteria in the intestine.^48^ While these data corroborate the importance of ethanolamine to AIEC fitness, some strains have lost the ability to utilize this metabolite due to deletions in some *eut* genes,^82^ highlighting the distinct adaptive routes of the different AIEC strains in the gut.

The metabolic plasticity of AIEC includes the consumption of short chain fatty acids (SCFAs),^28^ an important class of metabolites that is cardinal to the maintenance of intestinal homeostasis.^84,85^ In addition to being the preferred energy source for colonocytes,^86^ SCFAs lower the inflammatory tone of the gut.^84^ For example, butyrate, and less efficiently propionate, can inhibit histone deacetylases promoting the differentiation of Foxp3^+^ T regulatory cells, known to suppress inflammation.^87,88^ Additionally, the binding of SCFAs to G-protein coupled receptors (GPCR) 43 and 109A was shown to modulate T cell differentiation.^89,90^ In this regard, propionate enhanced the expression of Foxp3 and IL-10 in murine T regulatory cells only when GPCR-43 was present.^90^ Similarly, the binding of butyrate to GPCR-109A is important to induce the differentiation of IL-10 producing T regulatory cells.^89^ Therefore, it is not surprising that low endogenous concentrations of SCFAs have been associated with gut inflammation.^91^ In line with their pro-inflammatory behavior, AIEC are more adept than commensal *E. coli* in consuming SCFAs as carbon source,^28^ potentially leading to a decreased abundance of these anti-inflammatory molecules *in vivo*. This can potentially explain why CD patients have less intestinal SCFAs than healthy individuals.^91–93^ While this can be driven by the reduced abundance of fiber-fermenting bacteria in the gut,^91^ it remains possible that AIEC can further deplete SCFAs through catabolism. Moreover, the role of SCFAs in promoting AIEC fitness extends beyond providing these bacteria with energy. Upon exposure to butyrate or propionate, AIEC strains display increased adhesion and invasion of the epithelial cells.^94^ This is due to the upregulation of flagellar gene expression, which is known to potentiate the invasiveness of AIEC.^28^ Similarly, propionate was shown to promote the virulence and persistence of AIEC in the gut.^95,96^ Interestingly, propionate did not increase the adhesion and invasion of epithelial cells by commensal *E. coli*, suggesting that this adaptation is unique to AIEC.^96^ However, the modulation of AIEC virulence by SCFAs was shown to be largely dependent on the gut biogeography.^97^ For example, flagellar motility is increased under pH and SCFA concentrations mimicking that of the ileum, while the inverse is observed with colon mimicry.^97^ Therefore, more work is required to uncover the influence of the different gut microenvironments on the pathoadaptation of AIEC. While the genetic determinants for host colonization by AIEC were recently identified,^48^ approaches with higher resolution are required to investigate how bacterial fitness varies across the gut landscape.

In addition to the competition for carbon and nitrogen sources, the battle for metals is equally important for the success of pathogens to colonize the gut.^98^ Since many metabolic enzymes require metals, like iron, as co-factors, their acquisition is essential for bacteria. Comparative genomics revealed that the genomes of AIEC strains are enriched in iron acquisition genes relative to commensals.^81^ This includes heme utilization genes and siderophores. Upon their secretion to the extracellular environment, siderophores bind iron with high affinity.^98^ Using dedicated transporters, bacteria can uptake the iron-bound siderophores and shuttle them to the cytoplasm, where iron is liberated. Transcriptomics revealed the upregulation of several siderophore synthesis and transport genes by AIEC in the intestinal tissues^48^ and inside macrophages.^53^ This includes Yersiniabactin, a stealth siderophore that can evade sequestration by Lipocalin-2, a host defense protein known to chelate other siderophores.^98,99^ Interestingly, Yersiniabactin was shown to be required for the induction of fibrosis by AIEC in *Il10*^−/−^ mice, suggesting that this siderophore has immunomodulatory roles.^100^ This is in line with other studies showing that siderophores can interfere with immune functions. For example, enterobactin can inhibit myeloperoxidase, the enzyme responsible for generating the potent anti-microbial agent, hypochlorous acid.^101^ In addition to Yersiniabactin, AIEC strains carry other stealth siderophores, like Salmochelin. While this siderophore is dispensable for AIEC fitness in the gut during homeostasis, it is key for the expansion of these bacteria during psychological stress,^102^ a disease modifier associated with CD flares.^103^ This conditional essentiality of siderophores sheds light on their niche-specific roles, providing an explanation for their apparent functional redundancy. Nevertheless, these data highlight the potential of siderophores as targets for novel therapeutics. In accord with this, host immunization with siderophore-based vaccines was shown to elicit a strong mucosal immune response that can quell AIEC expansion and decrease gut inflammation.^104^ Taken together, these results underpin the potential of targeting iron acquisition systems as a strategy to complement the host nutritional immunity against CD-associated pathogens.

## The role of biofilms in promoting the persistence of AIEC

By embedding themselves in a self-produced extracellular matrix, bacteria can form biofilms on abiotic and biotic surfaces.^105–107^ The biofilm matrix constitutes proteins, exopolysaccharides, and DNA, which collectively protect the enclosed bacteria from external threats, including the immune cells.^108^ Indeed, the comparison of the spatial organization of bacteria during health and disease suggests that biofilm formation contributes to disease development. Using microscopy, Swidsinski *et al* showed that bacterial biofilms are more abundant in the mucosa of CD patients relative to healthy individuals by approximately two orders of magnitude.^109^ These dense biofilms were detected in the guts of 95% of the IBD patients, while only 35% of the healthy individuals had mucosal biofilms. Whether these biofilms are populated by healthy members of the microbiome or pathobionts remains undetermined. However, this triggered interest in exploring the ability of CD-associated pathogens, like AIEC, to form biofilms. In this regard, the archetypal AIEC strain LF82 was shown to form biofilms on the surface of intestinal epithelial cells, a process that requires the σ^E^ stress response. A strain overexpressing anti- σ^E^ factors formed less biofilms on epithelial cells. However, this strain displays impaired adhesion to host cells, making the direct contribution of σ^E^ to biofilm formation challenging to quantify.^110^ A recent study has identified biofilm formation as one of the key elements of AIEC fitness in the gut.^48^ This process requires a functional type IV secretion system (T4SS), which is employed by AIEC to form microcolonies on the epithelial surface. T4SS in bacteria comprises inner and outer membrane complexes that elaborate pili, which function in bringing the mating bacteria to proximity to allow DNA transfer.^111–113^ Although traditionally known for its role in conjugation, T4SS facilitates biofilm formation by *E. coli* both *in vitro*^114^ and *in vivo*.^48^ The enrichment of T4SS-encoding genes in the microbial metagenomes of CD patients suggests a role in disease development, potentially through promoting the ability of pathobionts to form biofilms and persist in the gut. CD is characterized by recurrent episodes of inflammation that are associated with bacterial expansion. One possible explanation for these flares is the presence of persistent bacterial communities that are shielded by their biofilms from the immune system. In the presence of specific environmental cues, these bacteria can then disseminate from their biofilms, launching a pro-inflammatory response in the gut, driving CD flares. In accord with this, biofilm production by AIEC was shown to be important for triggering inflammation in colitogenic mouse models.^115^ In addition to forming biofilms on mucosal surfaces, some AIEC strains like LF82 are able to form intracellular biofilm-like structures.^53^ During late stages of infection, the intracellular AIEC population induces a suite of genes required for biofilm formation inside the phagosomal compartment of macrophages. These intracellular biofilms were found to be pivotal for the survival of AIEC inside macrophages, which is a key trait that discriminates these bacteria from commensal *E. coli*.^22,51^

## Conclusions and future directions

Given the multifactorial nature of CD, it is challenging to fulfill Koch’s postulates when studying the contribution of microbes to its etiology. The perpetual complexity of the gut ecosystem, together with the influence of host genetics and environmental factors on the microbiome, limit our ability to decipher the role of particular organisms in driving inflammation. Traditionally, microbes have been viewed as a dichotomy of pathogens and commensals. Nevertheless, it is clear now that virulence can spawn within susceptible hosts as an emergent property.^33^ The AIEC group has received significant attention due to the high association of these strains with the CD environment^18,19^ and their ability to trigger inflammation in preclinical models.^30,32^ However, the lack of a clear evolutionary origin for this group suggests that the adherent-invasive phenotype might be an emergent property in the susceptible hosts.^116^ The isolation of AIEC from healthy individuals, while less frequently compared to CD patients, suggests that their pathogenesis is contextual. In line with this hypothesis, experimental evolution showed that the host environment selects for several pathoadaptive traits that promote the ability of AIEC to adhere to and invade the host cells.^28^ Similarly, the juxtaposition of host susceptibility and pathobiont evolution can lead to the emergence of detrimental microbial traits among commensals.^117^ Indeed, it is important to study the biochemical mechanisms underlying the virulence of AIEC. Nevertheless, it is equally important to unearth the triggers of emergent virulence among gut bacteria to develop a better understanding of CD etiology. This is critical to guide the drug screening pipelines aiming at developing selective therapies that can eradicate CD-associated pathobionts while maintaining the healthy microbiome.

## Data Availability

Data sharing is not applicable to this article as no new data were created or analyzed in this study.
